# Interleukin-31 expression and relation to disease severity in human asthma

**DOI:** 10.1038/srep22835

**Published:** 2016-03-09

**Authors:** Tianwen Lai, Dong Wu, Wen Li, Min Chen, Zhennan Yi, Dan Huang, Zhiliang Jing, Yingying Lü, Quanchao Lv, Dongming Li, Bin Wu

**Affiliations:** 1 Department of Respiratory and Critical Care Medicine, Affiliated Hospital, Institute of Respiratory Diseases, Guangdong Medical College, Zhanjiang, China; 2 Department of pathology, Affiliated Hospital, Guangdong Medical College, Zhanjiang, China

## Abstract

Interleukin 31 (IL-31) is a novel T helper type 2 effector cytokine that plays an important role in the pathogenesis of allergic diseases. However, its role in human asthma remains unclear. The aim of this study was to measure IL-31 levels in the serum, bronchoalveolar lavage fluid (BALF) and bronchial tissue of asthmatics and healthy subjects, and identify its possible correlation to disease severity. We quantified IL-31 levels in the serum of patients with asthma (n = 44), as well as in controls (n = 22). Of these subjects, 9 asthmatics and five controls underwent bronchoscopy with endobronchial biopsy and BALF collection. Our data showed that serum and BALF IL-31 levels were significantly elevated in patients with asthma compared with controls. Expressions of IL-31 and IL-31 receptor (IL-31RA and OSMR) were more prominent in the bronchial tissue in severe compared to mild asthma and controls. Serum IL-31 levels correlated positively with Th2 related cytokines (IL-5, IL-13, and TSLP), asthma severity or total serum immunoglobulin E (IgE), and inversely with asthma control and the forced expiratory volume in 1 second (FEV_1_). The current data may provide insight into the underlying pathogenesis of asthma, in which IL-31 has an important pathogenic role.

Asthma is characterized by airway inflammation, reversible airflow obstruction, persistent airway hyper-reactivity (AHR) and airway remodeling. Unlike other inflammatory diseases, the inflammatory response in asthma is associated with increased T help (Th) 2 cytokine production from T lymphocyte infiltration, including interleukin (IL)-4, IL-5 and IL-13^1^. Asthma is a serious global health problem affecting all age groups. Although some countries have seen a decline in hospitalizations and deaths from asthma, asthma still imposes an unacceptable burden on health care systems^2^. Despite recent guidelines focus on asthma control, asthma remains poorly controlled in many patients even under specialist care^3^. The outcomes might have been improved by earlier diagnosis and better monitoring. Thus, it is urgent to find new biomarkers to measure and monitor the amount of inflammation within the lungs of a patient with asthma and, as a result of better treatment of the disease. Recently, several studies reported on prominent functions of the novel T-cell-derived pro-inflammatory cytokine IL-31.

IL-31 belongs to the family of IL-6 cytokines that is expressed in kinds of human tissues^4^. IL-31 is produced by activated CD4^+^ T cells, mainly from the Th 2 subset^4^. The activity of human IL-31 is mediated through a receptor complex composed of IL-31 receptor A (IL-31RA) and oncostatin M receptor (OSMR)^1^. Binding of IL-31 to its receptor activates Jak/STAT, PI3K/AKT, p38 mitogen-activated protein kinases (MAPK), extracellular signal-regulated kinase (ERK) and c-Jun N-terminal kinase (JNK) pathways^5^. Recent studies indicate that IL-31 plays an important role in the induction of chronic inflammation and regulates various processes of innate and adaptive immunity in tissues that are exposed to the environment^1^,^6^. Increased IL-31 serum levels have been observed in different skin diseases as well as in inflammatory diseases, such as Crohn disease and ulcerative colitis^1^,^7^,^8^,^9^,^10^,^11^,^12^. Moreover, IL-31 stimulates secretion of proinflammatory cytokines, and matrix metalloproteinases^1^,^13^,^14^. Recent evidence has indicated that IL-31 might be involved in promoting allergic inflammation and an airway epithelial response that may characterize allergic asthma^15^,^16^,^17^. In patients with asthma, the IL-31 single nucleotide polymorphisms (SNPs) were significantly correlated with total serum levels of IgE^18^. IL-31 has also been shown to significantly increase epidermal growth factor (EGF) in a transformed human bronchial epithelial cell line^17^. However, there are no studies investigating the expression pattern of IL-31 in patients with asthma of varying severity in a clinical setting.

In the present study, it was hypothesised that the upregulation of IL-31 expression is more pronounced in more severe forms of asthma. Levels of IL-31 in the serum, bronchoalveolar lavage fluid (BALF) and bronchial tissue of mild-to-moderate and severe asthma patients were determined and compared to those of healthy controls. It was also examined whether or not correlations exist between IL-31 expression and disease severity (e.g., lung function, asthma control).

## Results

### Clinical data

There was no significant difference in age, gender or body mass index (BMI). The total IgE levels and the percentage of peripheral blood eosinophils in patients with asthma were higher than those in the controls. Compared with patients in the control group, those in the asthma group had more severely compromised lung function. Twenty-three patients (52.3%) had at least one positive SPT result. Sensitizations to house dust mites and cockroaches were the most common. The characteristics of participants were summarized in Table 1.

### IL-31 levels in the serum and BALF

**Figure 1:**
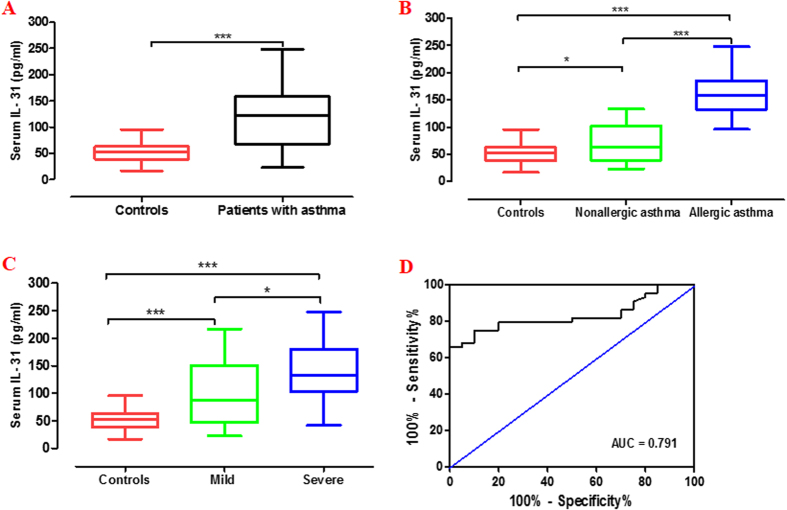
Serum IL-31 levels in patients with asthma and controls. The levels of serum IL-31 were assessed in patients with asthma (n = 44) and controls (n = 22) as measured by ELISA. (**A**) Serum IL-31 was increased in patients with asthma compared with the controls; (**B**) When patients were stratified according to the status of atopy, the serum IL-31 levels in patients with allergic asthma (n = 23) were higher than those in patients with nonallergic asthma (n = 21) and controls; (**C**) Serum IL-31 was increased in severe asthma patients (n = 22) compared with mild asthma patients (n = 22) and controls; (**D**) Receiver operating characteristic (ROC) curve for distinguishing patients with asthma from the controls. The area under the ROC curve was 0.791 (95% CI: 0.66 to 0.86). Data are presented as median with interquartile range. *p < 0.05, ***p < 0.001.

**Figure 2:**
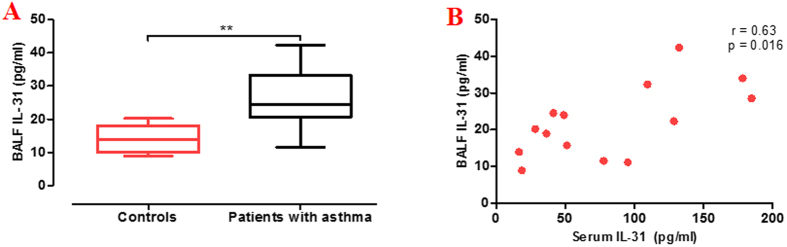
IL-31 levels in the bronchoalveolar lavage fluid (BALF). (**A**) BALF samples were measured by ELISA and the BALF IL-31 levels in patients with asthma (n = 9) were significantly increased compared with the controls (n = 5); (**B**) Spearman’s rank correlation analysis showed the serum IL-31 levels correlated positively with BALF IL-31 levels (*r* = 0.63; p = 0.016). Data are presented as median with interquartile range. **p < 0.01.

The serum IL-31 levels in patients with asthma were higher than those in the controls (median 122.6 [66.2–158.1] pg/ml *versus* 52.5 [38.5–62.7] pg/ml; p < 0.001, Fig. 1A). When patients were stratified according to the status of atopy, the serum IL-31 levels in patients with allergic asthma were higher than those in patients with nonallergic asthma (157.7 [132.5–184.7] pg/ml *versus* 63.4 [39.1–102.3] pg/ml; p < 0.05) and controls (157.7 [132.5–184.7] pg/ml *versus* 52.5 [38.5–62.7] pg/ml; p < 0.001) (Fig. 1B). When patients were stratified according to disease severity, we found that the serum IL-31 levels for patients with severe asthma were higher than those in mild asthma (133.0 [103.0–180.1] pg/ml *versus* 86.8 [46.4–150.5] pg/ml; p = 0.011) and those in the controls (133.0 [103.0–180.1] pg/ml *versus* 52.5 [38.5–62.7] pg/ml; p < 0.001) (Fig. 1C). The ideal cutoff point for distinguishing patients with asthma from those in the controls was 62.9 pg/ml (sensitivity, 80.0%; specificity, 79.5%; AUC, 0.791; 95% CI: 0.66 to 0.86) (Fig. 1D). Moreover, IL-31 levels were significantly increased in the BALF of patients with asthma compared with the controls (Fig. 2A). The serum IL-31 levels correlated positively with BALF IL-31 levels (*r* = 0.63; p = 0.016) (Fig. 2B).

### Serum IL-31 levels in relation to Th2 related cytokines

**Figure 3:**
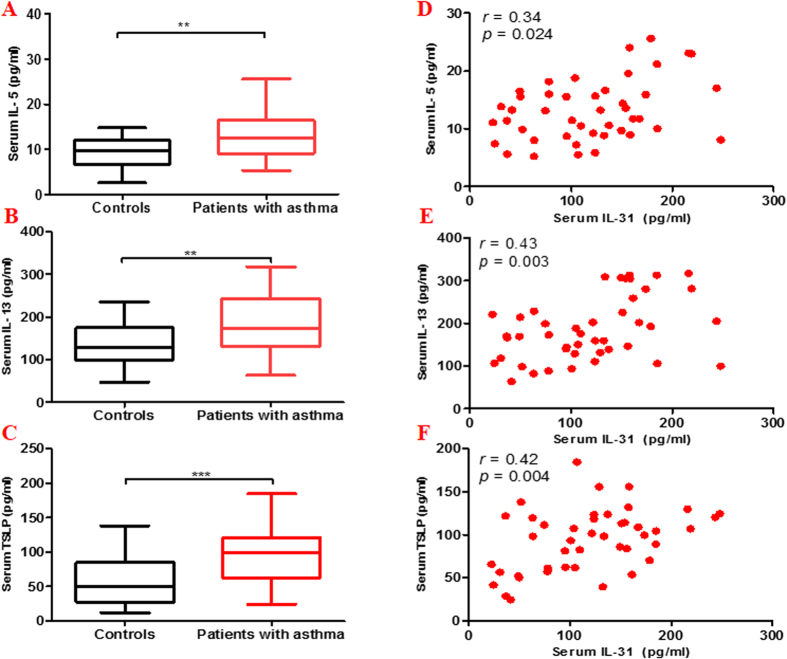
Serum IL-31 levels in asthmatic patients correlate with the Th2 cytokines. The classical Th2 cytokines such as IL-5, IL-13 and thymic stromal lymphopoietin (TSLP) were further measured by ELISA. The levels of serum IL-5, IL-13 and TSLP were significantly higher in patients with asthma (n = 44) compared with control subjects (n = 22) (**A–C**). Spearman’s rank correlation analysis showed a significant correlation between the serum IL-31 levels and the serum IL-5 levels (*r* = 0.34, p = 0.024), the serum IL-13 levels (*r* = 0.43, p = 0.003), the serum TSLP levels (*r* = 0.42, p = 0.004) (**D–F**). Data are presented as median with interquartile range. **p < 0.01, ***p < 0.001.

IL-31 is a Th2 cytokine, to determine whether its serum concentration is correlated with other asthma related Th2 cytokines in asthma, we further measured the Th2 related cytokines IL-5, IL-13 and thymic stromal lymphopoietin (TSLP). We found that the serum levels of IL-5, IL-13 and TSLP were significantly elevated in patients with asthma compared with controls (Fig. 3A–C). In addition, Spearman’s rank correlation analysis revealed that serum IL-31 levels correlated positively with the serum levels of IL-5, IL-13 and TSLP in asthmatic patients (IL-31/IL-5, *r* = 0.34; IL-31/IL-13, *r* = 0.43; IL-31/TSLP, *r* = 0.42, respectively) (Fig. 3D–F).

### Serum IL-31 levels in relation to asthma control

**Figure 4:**
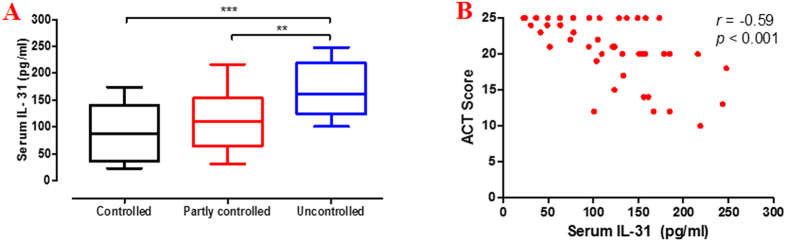
Serum IL-31 levels in relation to asthma control. Patients were classified into three groups based on ACT scores. Patients with uncontrolled asthma (n = 15) had statistically higher serum IL-31 levels than patients with partly controlled (n = 18) and completely controlled asthma (n = 11) (**A**). The correlation between serum IL-31 levels and ACT score was assessed using Spearman’s rank correlation analysis (**B**). Data are presented as median with interquartile range. **p < 0.01, ***p < 0.001.

Fifteen patients (34.1%) were completely controlled, 18 (40.9%) were partly controlled, and 11 (25.0%) were uncontrolled (Table 1). The mean ACT score was 20.7 ± 0.5. Patients with uncontrolled asthma had statistically higher serum IL-31 levels than patients with partly controlled (*p* < 0.01) and completely controlled asthma (*p* < 0.001) (Fig. 4A). The serum IL-31 levels negatively correlated with ACT score (*r* = −0.59, p < 0.001) (Fig. 4B).

### Serum IL-31 levels in relation to other clinical parameters

**Figure 5:**
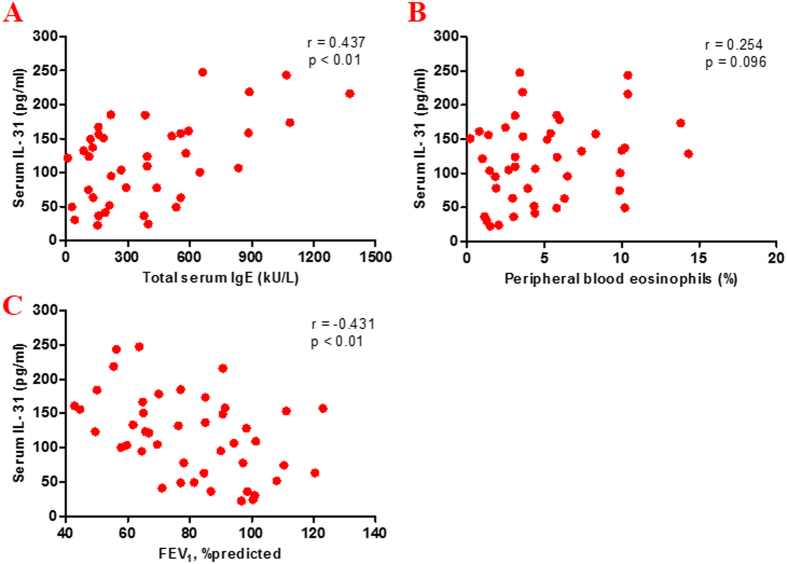
Serum IL-31 levels in relation to other clinical parameters. Spearman’s rank correlation analysis showed a significant correlation between the serum IL-31 levels. (**A**) The total serum immunoglobulin (Ig) E levels (*r* = 0.437, p < 0.01); (**B**) forced expiratory volume in 1 s (FEV_1_)/predicted value (*r* = −0.431, p < 0.01); (**C**) There was no significantly relationship between serum levels of IL-31 and percentage (%) of peripheral blood eosinophils (*r* = 0.254, p = 0.096).

Spearman’s rank correlation analysis showed that serum IL-31 levels in patients with asthma correlated positively with serum total IgE levels and negatively with FEV_1_ (*r* = 0.437, p < 0.01 and *r* = −0.431, p < 0.01, respectively), but not with the percentage of peripheral blood eosinophils (*r* = 0.254, p = 0.096) (Fig. 5). There were no significant correlations between serum IL-31 levels and other clinical parameters such as age, FEV_1_/FVC.

### Immunohistochemical analysis

**Figure 6:**
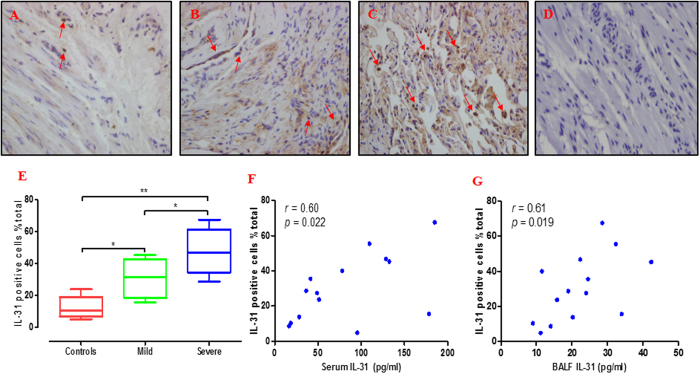
Immunohistochemical analysis of IL-31. Bronchial tissue obtained from healthy subjects (n = 5) and asthmatic patients (n = 9) who underwent fibreoptic bronchoscopy was processed immunohistochemically to show tissue IL-31 expression. Specific staining for IL-31 is brown (red arrows), whereas nuclei are stained blue (Magnification × 400). Representative photomicrographs are presented from (**A**) a healthy individual and (**B**) a patient with mild asthma and (**C**) patient with severe asthma and (**D**) the corresponding isotypic control. The expression of IL-31 in patients with severe asthma were higher than those in controls and paitents with mild asthma (**E**) Moreover, the percentage (%) of cells expressing IL-31 correlated positively with serum IL-31 levels (*r* = 0.60; p = 0.022, (**F**)) and BALF IL-31 levels (*r* = 0.61; p = 0.019, (**G**)). Data are presented as median with interquartile range. *p < 0.05, **p < 0.01.

**Figure 7:**
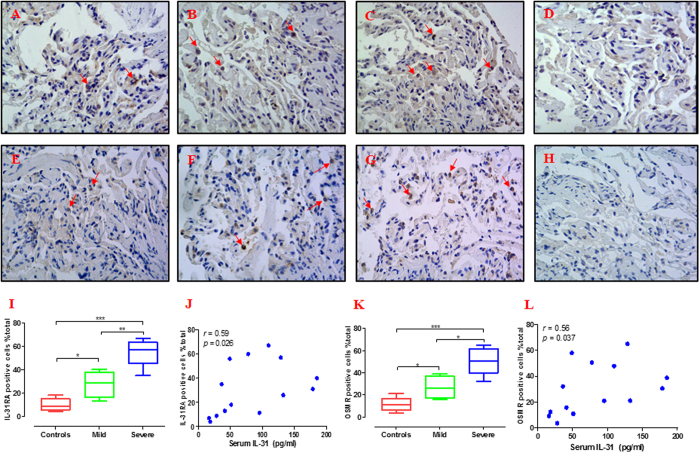
Immunohistochemical analysis of IL-31 receptor (IL-31RA and OSMR). Immunohistochemical staining was performed to assess the expression of IL-31RA (**A–D**) and OSMR (**E–H**). (Magnification × 400, red arrows). Representative photomicrographs are presented from a healthy individual (**A,E**), patient with mild asthma (**B,F**), patient with severe asthma (**C,G**) and the corresponding isotypic control (**D,H**). The expression of IL-31RA and OSMR in patients with severe asthma were higher than those in controls and patients with mild asthma (**I,K**). Moreover, the relationships between the serum IL-31 levels and percentage (%) of cells expressing of IL-31RA and OSMR were assessed by Spearman’s rank correlation analysis (**J,L**). Data are presented as median with interquartile range. *p < 0.05, **p < 0.01, ***p < 0.001.

Quantification of lung IL-31 expression revealed that the percentage of IL-31-positive cells in patients with severe asthma (46.8% [34.3–61.5%]) was significantly greater than the percentages found in the patients with mild asthma (31.5% [18.6–42.8%]; p < 0.05) and the controls (10.4% [6.8–18.8%]; p < 0.01) (Fig. 6A–C,E). No immunostaining was observed in control isotype rabbit IgG-treated tissue sections (Fig. 6D). In addition, the percentage (%) of total cells correlated positively with serum IL-31 levels (*r* = 0.60; p = 0.022) and BALF IL-31 levels (*r* = 0.61; p = 0.019) (Fig. 6F–G). In order to make a solid conclusion of the crucial roles of IL-31 in asthma, we further assessed the IL-31 receptor IL-31RA and OSMR in the tissues. We found that faint expression of IL-31RA and OSMR in the controls (Fig. 7A,E) and there were more positive cells evident in the patients with severe asthma (Fig. 7C,G) compared with patients with mild asthma (Fig. 7B,F). No immunostaining was observed in control isotype rabbit IgG-treated tissue sections (Fig. 7D,H). Semi quantitative analysis of expression of IL-31RA and OSMR in lung sections were performed (Fig. 7I,K). Spearman’s rank correlation analysis showed the percentage (%) of cells expressing of IL-31RA and OSMR correlated positively with serum IL-31 levels (*r* = 0.59; p = 0.026 and *r* = 0.56, p = 0.037, respectively) (Fig. 7J,L).

## Discussion

In the present study, we explored the potential role of IL-31 measurement in the management of asthma. Our study indicated that serum IL-31 levels were increased in patients with asthma, as compared with control subjects, and correlated with the severity of asthma. Moreover, the serum IL-31 levels correlated positively with the level of expression of IL-31 and IL-31 receptor (IL-31RA and OSMR) in the airway, Th2 related cytokines (IL-5, IL-13, and TSLP), clinical parameters of disease severity and correlated inversely with lung function. These results suggested that IL-31 plays an important role in determining the pathobiologic characteristics of severe asthma.

Asthma is described as a chronic inflammatory disorder of the conducting airways, typically associated with aberrant Th2 activation and response against environmental antigens. Cytokines play a key role in the host response to immunological challenges. IL-31 was expressed by all of the Th2 clones and not by Th1, Th17, or Th22. This expression was dependent on autocrine IL-4 expression from these clones because it could be reduced if blocking antibodies to IL-4 were present. Interestingly, Th1 clones were able to express IL-31 if IL-4 was added to culture^19^. Recently, IL-31 has been reported to be involved in the development of itch, making the estimation of itch intensity a future reality^20^. IL-31 has been described to play a key role in the pathogenesis of atopic dermatitis. Serum IL-31 levels were significantly higher in patients with atopic dermatitis (AD) compared with those in nonatopic healthy control subjects^8^,^21^. Cheung *et al.* showed that IL-31 could significantly induce the release of pro-inflammatory cytokines IL-1beta, IL-6 and AD-related chemokines CXCL1, CXCL8, CCL2 and CCL18 from eosinophils, via functional cell surface IL-31 receptor^10^. Further studies have demonstrated that IL-31 may be involved in promoting allergic inflammation and triggering airway epithelial responses such as allergic asthma^16^,^22^. IL-31 regulates the gene and protein expressions of epidermal growth factor (EGF), vascular endothelial growth factor (VEGF) and monocyte chemoattractant protein-1 (MCP-1/CCL2) in human bronchial epithelial cells^17^. We found that circulating levels of IL-31 in the moderate-to-severe and uncontrolled groups were significantly higher than those in the mild and controlled groups, which suggested that serum levels of IL-31 correlate with asthma severity. IL-31 levels may therefore be a useful adjunct in the objective identification and management of severe asthma.

IL-31 is known to associate with Th2 cytokines (e.g., IL-4, IL-5, and IL-13) and a possible interaction between IL-31 and Th2 inflammation has been suggested^1^. We further measured the Th2 related cytokines (IL-5, IL-13 and TSLP). As expected, we found that the levels of IL-5, IL-13 and TSLP were higher in asthmatic patients than those in the controls. Moreover, IL-31 expression was correlate with the expression of IL-5, IL-13 and TSLP. This is consistent with previous studies by Neis *et al.*^23^. In the latter, IL-31 expression has been shown to correlate with the expression of the Th2 cytokines IL-4 and IL-13 in allergic contact dermatitis. IL-31 signals through the heterodimeric receptor IL-31RA and OSMR, and has been linked with the development of atopic dermatitis^21^. The regulatory role of IL-31R signaling is limited to type 2 responses. IL-31R signaling as a novel negative regulatory pathway that specifically limits type 2 inflammation. IL-31RA–deficient mice produce increased levels of OSM-inducible cytokines during airway sensitization and challenge^13^. To make a solid conclusion of the crucial roles of IL-31 in asthma, the expressions of IL-31RA and OSMR in the tissues were further assessed. Our data showed that expressions of IL-31RA and OSMR in lung sections were more pronounced in more severe forms of asthma and positively correlated with serum IL-31 levels. Collectively, these data further confirmed that the levels of IL-31 is useful indicator for asthma.

Severe exacerbation of asthma is major concern, as it is responsible for the mortality associated with asthma, and contributes heavily to levels of mortality and health costs of the disease^24^. Current evidence suggests that an estimated 57% of adolescent and adult respondents had uncontrolled asthma and a substantial proportion of severe cases were attributable to allergic immunoglobulin E (IgE)-mediated mechanisms. Yu *et al.* showed that the IL-31 SNPs were significantly correlated with total serum levels of IgE in patients with asthma^18^. Our data showed that the serum IL-31 levels correlated positively with total serum IgE levels and correlated inversely with FEV_1_. Together, these findings indicated that IL-31 induced a higher expression of Th2 cytokines, which induced more severe Th2 inflammation and aggravating clinical symptoms.

Eosinophilic asthma has been the focus of recent clinical trials using biologicals. A recent study showed that eosinophilic asthma is associated with CCL-26 and revealed that CCL-26 as a potential target for treating patients with eosinophilic asthma that are refractory to classic therapies^25^. Thus, we also tried to perform subgroup analysis base on the percentage of peripheral blood eosinophils (normal range: 0.5–5%). We divided asthmatic patients into two groups: Eos ≤ 5% and Eos > 5%. We found that there were no significantly difference between two groups which might be due to the small sample size, although there was a trend to higher median levels in patients with Eos > 5% (n = 19) compared with patients with Eos ≤ 5% (n = 25) (median, 133.5 pg/ml vs 104.9 pg/ml, p = 0.0941) (Supplemental Fig. 1). Although our finding suggest that IL-31 may be useful indicator for asthma, future studies with larger sample sizes as well as animal experiments and *in vitro* experiments should be performed to determine whether IL-31 can be used as a potential drug target for treating asthmatic patients. Moreover, perspective studies will also be required to determine whether IL-31 levels are stable or increase during exacerbations of asthma and whether the levels of IL-31 would decline when those exacerbation subjects are completely controlled after treatments.

In summary, we have shown that IL-31 is found in increased quantities in the serum and lungs of patients with asthma, in whom the protein levels correlate positively with the severity of disease and total IgE and inversely with lung function. The present study provides new knowledge about the potential role of IL-31 in asthma that contributes to further understanding of its pathophysiology.

## Methods

### Study population

A total of 44 asthmatic patients (aged 44.5 ± 2.3 years) participated in the present study. We also recruited 22 non-atopic healthy volunteers (aged 38.6 ± 1.8 years) with normal spirometry from the communities surrounding our hospital to serve as controls. From this population, 9 patients with steady-state asthma and five healthy controls consented to undergo research fibreoptic bronchoscopy with BALF collection and endobronchial biopsy when possible. Serum samples were collected from all participants.

Asthma was diagnosed according to the Global Initiative for Asthma (GINA) guidelines based on a history of recurrent episodes of wheezing and chest tightness, with or without cough, and impaired spirometry with reversibility in FEV_1_ of >12% and 200 ml after salbutamol administration or hyperresponsiveness to inhaled methacholine^2^. Exclusion criteria were the following: oral corticosteroids or respiratory tract infection within the preceding four weeks prior to enrolment; any chronic cardiopulmonary disease other than asthma (including COPD); pregnant. Healthy individuals had no history of asthma or any other chronic disease. They were free of respiratory tract infection in the four weeks prior to the study. All subjects were nonsmokers.

The corresponding protocol was approved by the Ethics of Research Committee of the Medical College of Guangdong. Informed and written consent was obtained from all participating subjects, which was in accordance with the Declaration of Helsinki.

### Pulmonary function tests

Spirometry was measured with standard spirometric techniques (Jaeger, Germany) at least 6 hours after a patient’s most recent treatment with albuterol, according to American Thoracic Society (ATS) guidelines^26^. Patients were divided into three groups on the basis of severity of asthma, according to GINA: (1) mild asthma (FEV_1_ ≥ 80%); (2) moderate asthma (FEV_1_ 60–79%); and (3) severe asthma (FEV_1_ < 60%)^2^.

### Asthma control test (ACT) and skin prick test (SPT)

Patients were classified into three groups based on ACT scores^2^: completely controlled (ACT score = 25), partly controlled (ACT score range = 20–24), and uncontrolled (ACT score range = 5–19). Atopy was tested with the SPT. Twelve common animal and aeroallergens (ALK-Abello, Horsholm, Denmark) were tested. The test was considered positive if the wheal diameter of at least 3 mm.

### Enzyme-linked immunosorbent assay (ELISA)

The levels of IL-31, IL-5, IL-13 and thymic stromal lymphopoietin (TSLP) were assessed by ELISA, using ELISA Kits for the assay of human IL-31, IL-5, IL-13 and TSLP (Uscn Life Science Inc. Wuhan) according to the manufacturer’s protocol. The minimum detection limit of the IL-31, IL-5, IL-13, and TSLP assay is 12.2 pg/ml, 2.7 pg/ml, 5.7 pg/ml and 5.7 pg/ml, respectively.

### Total IgE and peripheral eosinophils (%)

Two milliliters of non-heparinized venous peripheral blood sample was placed at room temperature for 30 min and then blood was centrifuged and the serum was stored at a temperature of −80 °C. Total serum IgE levels were detected once by a fluoroenzyme immunoassay in clinical laboratory (Beckman Coulter image 800 specific protein analysis system, USA) from patients with asthma and controls. The normal reference range of total serum IgE was less than 165 kU/L. Three milliliters of heparinized venous peripheral blood sample was collected from each patient and control subject. The percentage of peripheral blood eosinophils was determined using the Beckman Coulter LH500 (USA), a quantitative and automated hematologic analyzer and leukocyte differential cell counter for *in vitro* diagnostic use in clinical laboratory.

### Sample collection

Bronchoscopy was performed according to the guidelines of the American Thoracic Society^27^. Bronchial biopsy specimens were obtained from the bifurcations of the right middle lobe or lower lobes following BALF collection. BALF samples were centrifuged for 15 min at 300 × *g* at 4 °C and were aliquoted and stored at −80 °C within 2 h after collection. There were no complications from the procedures.

### Immunohistochemistry

The slides were incubated with either a rabbit polyclonal Ab against IL-31 (Proteintech, Chicago, USA), IL-31RA, OSMR (Uscn Life Science Inc. Wuhan) or a rabbit IgG (Abcam, Cambridge, UK) at the same concentration as the control isotype. The immunoreaction was visualized using a 3,3-diaminobenzidine chromogen solution (DAB substrate kit, Abcam, Cambridge, UK) according to the manufacturer’s instructions. Quantitative measurements of IL-31-positive cells in the bronchial tissue were performed as previously described^28^. Briefly, IL-31-positive and -negative cells were counted in each biopsy specimen. Bronchial cells positive for the IL-31 antibody were expressed as a percentage of total cells. Sections were examined using a light microscope (BX51; Olympus, Japan) and quantified by the Image Pro 6.1 software (Media Cybernetics). The intraobserver error was assessed by performing three separate counts of the same section on different occasions.

### Statistical analysis

Data were expressed as median with interquartile range, unless otherwise stated. Kolmogorov-Smirnov (KS) test was performed to examine normality of distribution. The values were compared among the study groups using either the Mann-Whitney U test or the Kruskal-Wallis test. Categorical data were compared using Pearson’s chi-squared test. The interrelationships between different parameters were determined using the Spearmans correlation. To determine whether IL-31 differentiated between healthy controls and asthma, the area under the curve (AUC) receiver operating characteristics (ROC) for IL-31 was analyzed. GraphPad Prism 5.0 software (GraphPad Software Inc., San Diego, CA, USA) was used for the analyses and graphs. Statistical significance was set at a p value < 0.05.

## Additional Information

**How to cite this article**: Lai, T. *et al.* Interleukin-31 expression and relation to disease severity in human asthma. *Sci. Rep.* **6**, 22835; doi: 10.1038/srep22835 (2016).

## Supplementary Material

Supplementary Information

## Figures and Tables

**Table 1:** Characteristic of the study participants.

Characteristic	Controls	Patients with asthma	p-value
Number, n	22	44	
Age, yrs	38.6 ± 1.8	44.5 ± 2.3	NS
Sex (F/M)	8/14	21/23	NS
BMI (kg/m^2^)	22.3 ± 1.1	21.7 ± 1.5	NS
FEV_1,_ % predicted	93.4 ± 1.2	80.3 ± 3.1	0.004
≥80%, n (%)	22 (100)	22 (50)	
60–79%, n (%)	0	14 (31.8)	
<60%, n (%)	0	8 (19.2)	
FEV_1_/FVC, %	90.8 ± 3.4	77.1 ± 0.8	< 0.001
Total IgE, kU/L	138.2 ± 46.5	403.9 ± 51.8	0.002
Eosinophil in WBC%	2.9 ± 0.4	5.1 ± 0.5	0.013
Atopy, n (%)	0	23 (52.3)	
House dust mites, n	–	9	
Cockroaches, n	–	5	
Cat, n	–	3	
Dog, n	–	3	
Ragweed, n	–	2	
Alternaria, n	–	1	
ACT score	NA	20.7 ± 0.7	
25, n (%)	NA	15 (34.1)	
20–24, n (%)	NA	18 (40.9)	
5–19, n (%)	NA	11 (25.0)	

Data are presented as mean ± SEM or n (%), unless otherwise stated.

Note: F, female; M, male; BMI, body mass index; FEV_1_, forced expiratory volume in one second; FVC, forced vital capacity; IgE, immunoglobulin; WBC, white blood cells; ACT, asthma control test; NS, not significant. NA, not assessed.
