# Supplementary material for: Interleukin-31 expression and relation to disease severity in human asthma
Source: Sci Rep. 2016 Mar 9;6:22835. doi: 10.1038/srep22835 (PMC4783779; doi:10.1038/srep22835)
Supplement: Supplementary Information [file srep22835-s1.pdf]

Title page

Interleukin-31 expression and relation to disease severity in human asthma

Tianwen Lai[1†]; Dong Wu[1†]; Wen Li[1†]; Min Chen[1]; Zhennan Yi[1]; Dan Huang[1]; Zhiliang Jing[2]; Yingying Lǘ[1]; Quanchao Lv[1]; Dongming Li[1*]; Bin Wu[1*]

1. Department of Respiratory and Critical Care Medicine, Affiliated Hospital, Institute of Respiratory Diseases, Guangdong Medicine College, Zhanjiang, China.
2. Department of pathology, Affiliated Hospital, Guangdong Medicine College, Zhanjiang, China.

[†]Equal contributors

*Correspondence to Bin Wu or Dongming Li. Email: wubin621011@126.com or 1335887835@qq.com.
Tel: 86-759-2387-413; Fax; 86-571-8778-3729

Supplementary information

Supplemental Fig. 1 Patients were stratified two groups according to the percentage of peripheral blood eosinophils: patients with Eos > 5% group (n = 19) and patients with Eos ≤ 5% group (n = 25). There were no significantly difference between two groups (median [range], 133.5 [95.7-173.5] pg/ml vs 104.9 [46.7-155.0] pg/ml, p = 0.0941).

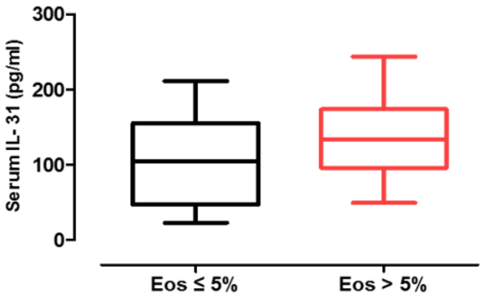

Supplemental Fig. 1
